# Supplementary material for: Deep sequencing of primary human lung epithelial cells challenged with H5N1 influenza virus reveals a proviral role for CEACAM1
Source: Sci Rep. 2018 Oct 19;8:15468. doi: 10.1038/s41598-018-33605-6 (PMC6195505; doi:10.1038/s41598-018-33605-6)

Deep sequencing of primary human lung epithelial cells challenged with H5N1 influenza virus reveals a proviral role for CEACAM1

Siying Ye[1,2*], Christopher J. Cowled[3], Cheng-Hon Yap[4], John Stambas[1,2]

[1]School of Medicine, Deakin University, Waurn Ponds, Victoria, Australia. [2]AAHL CSIRO Deakin Collaborative Biosecurity Laboratory, East Geelong, Victoria, Australia. [3]Health & Biosecurity, CSIRO, East Geelong, Victoria, Australia. [4]University Hospital Geelong, Barwon Health, Geelong, Victoria, Australia.

Correspondence and requests for materials should be addressed to S.Y. (email: siying.ye@csiro.au).

Table S5. qRT-PCR primers and pairing for the detection of 4 *CEACAM1* human variants.

**Primers**	**Sequence**
1-4Fwd	ACC CTG TCA AGA GGG AGG AT
1-LRev	TGA GGG TTT GTG CTC TGT GA
1-3Fwd	TAG TCA CTG ATA ATG CTC TAC C
1-SRev	GTC CTG AGC TGC CGG TCT

Reverse / Forward	1-LRev	1-SRev
1-4Fwd	1-4L	1-4S
1-3Fwd	1-3L	1-3S

Figure 3C: A representative Western blot of three individual experiments and protein band density analysis of endogenous CEACAM1 protein expression in A549 cells following infection with PR8 virus at MOIs of 2, 5 and 10 at 24 and 48 hpi.

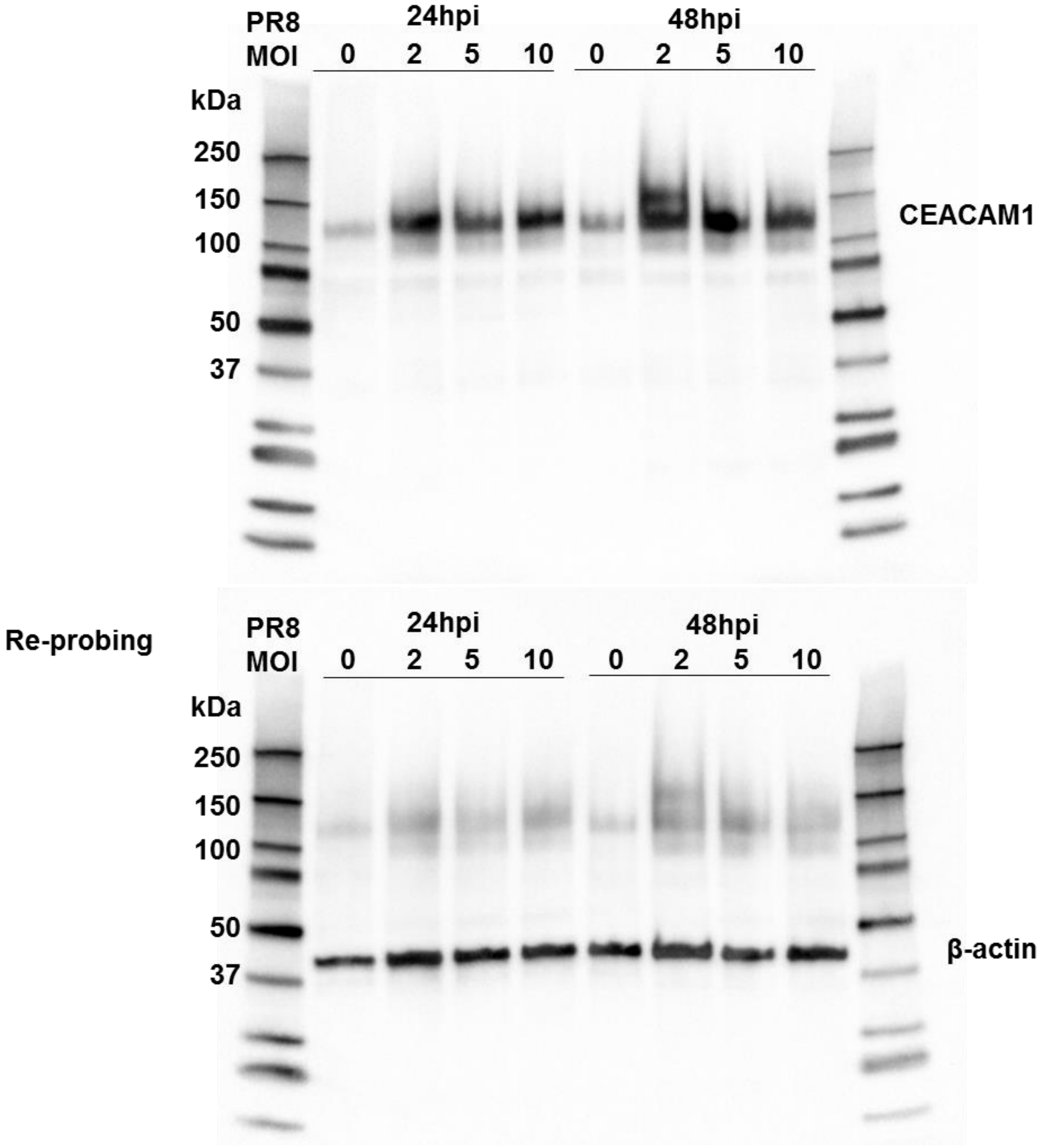

Figure 3D: A representative Western blot of three individual experiments and protein band density analysis of endogenous CEACAM1 protein expression in primary human ATII cells infected with PR8 or HPAI H5N1 virus at various MOIs and time points as indicated.

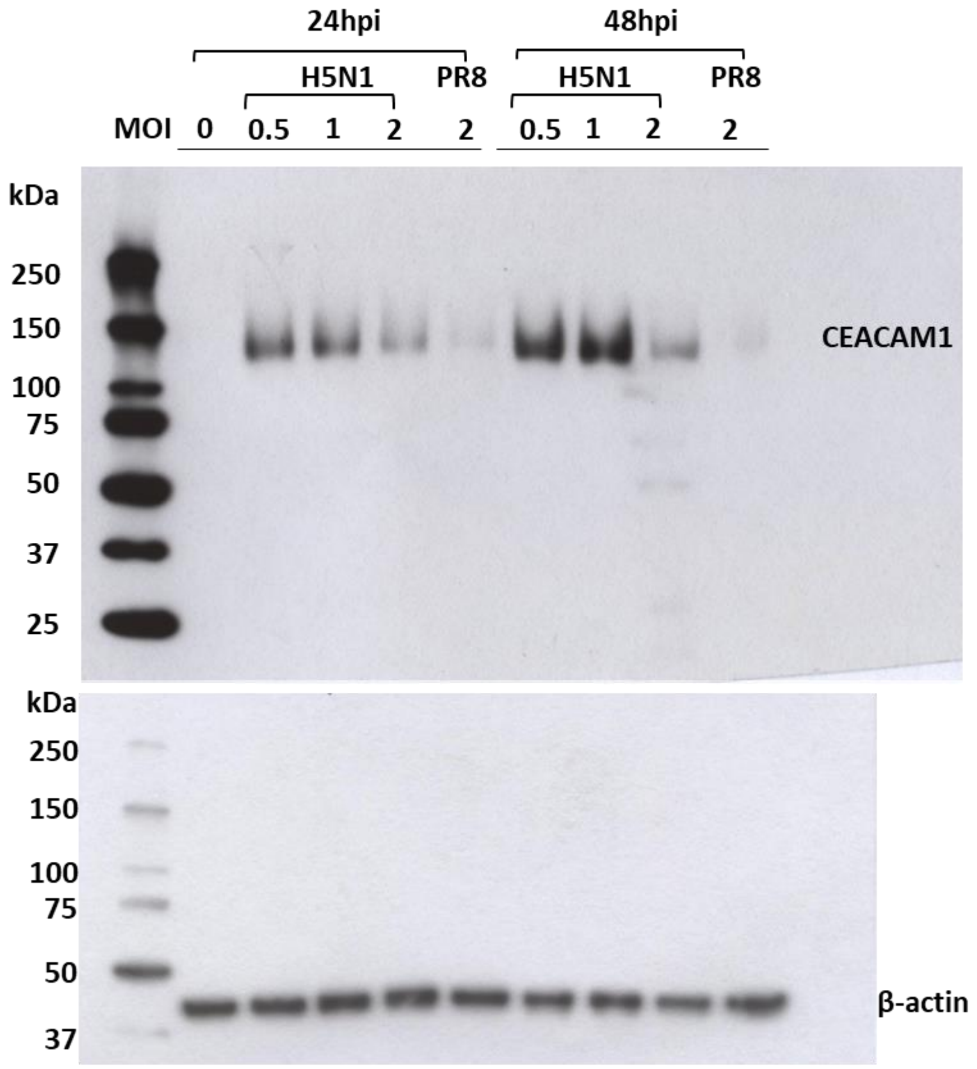

Figure 4B: ATII

A representative Western blot of three individual experiments and protein band density analysis of siCEACAM1-mediated knockdown of endogenous CEACAM1 in ATII transfected with siCEACAM1 in comparison to mock-transfected cells and cells transfected with siNeg control.

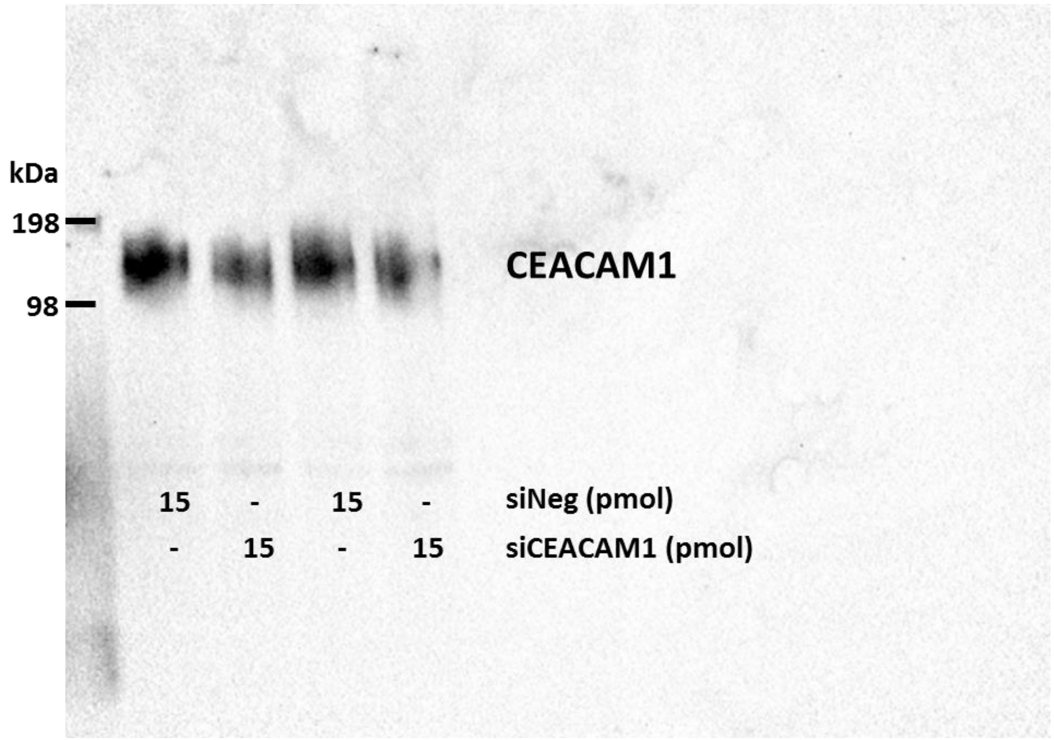

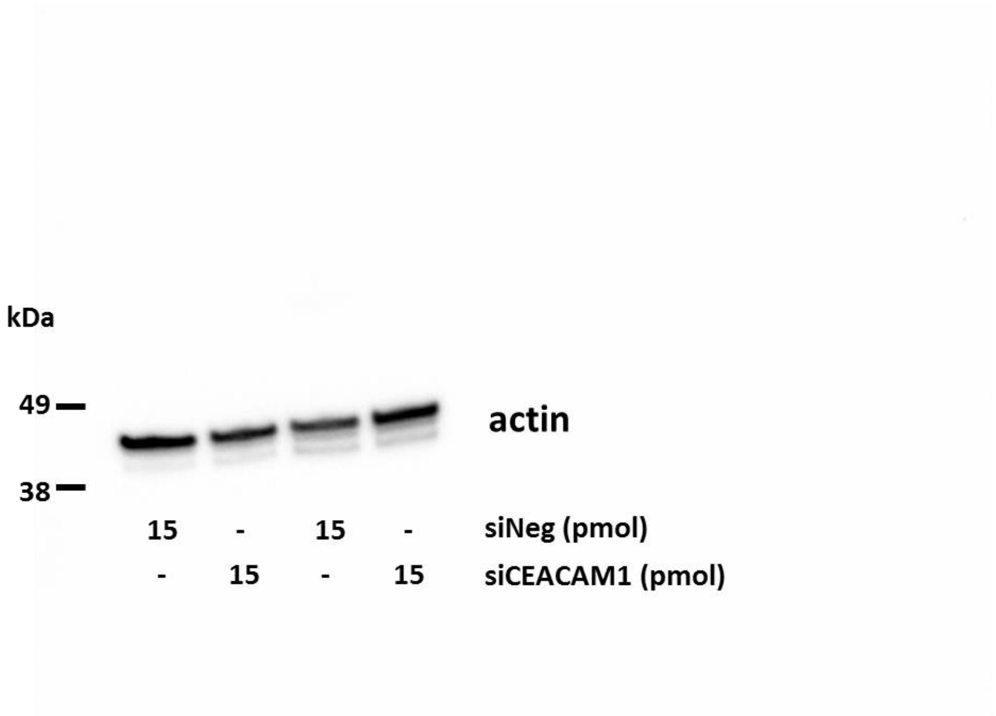

Figure 4B: A549

A representative Western blot of three individual experiments and protein band density analysis of siCEACAM1-mediated knockdown of endogenous CEACAM1 in A549 cells transfected with siCEACAM1 in comparison to mock-transfected cells and cells transfected with siNeg control.

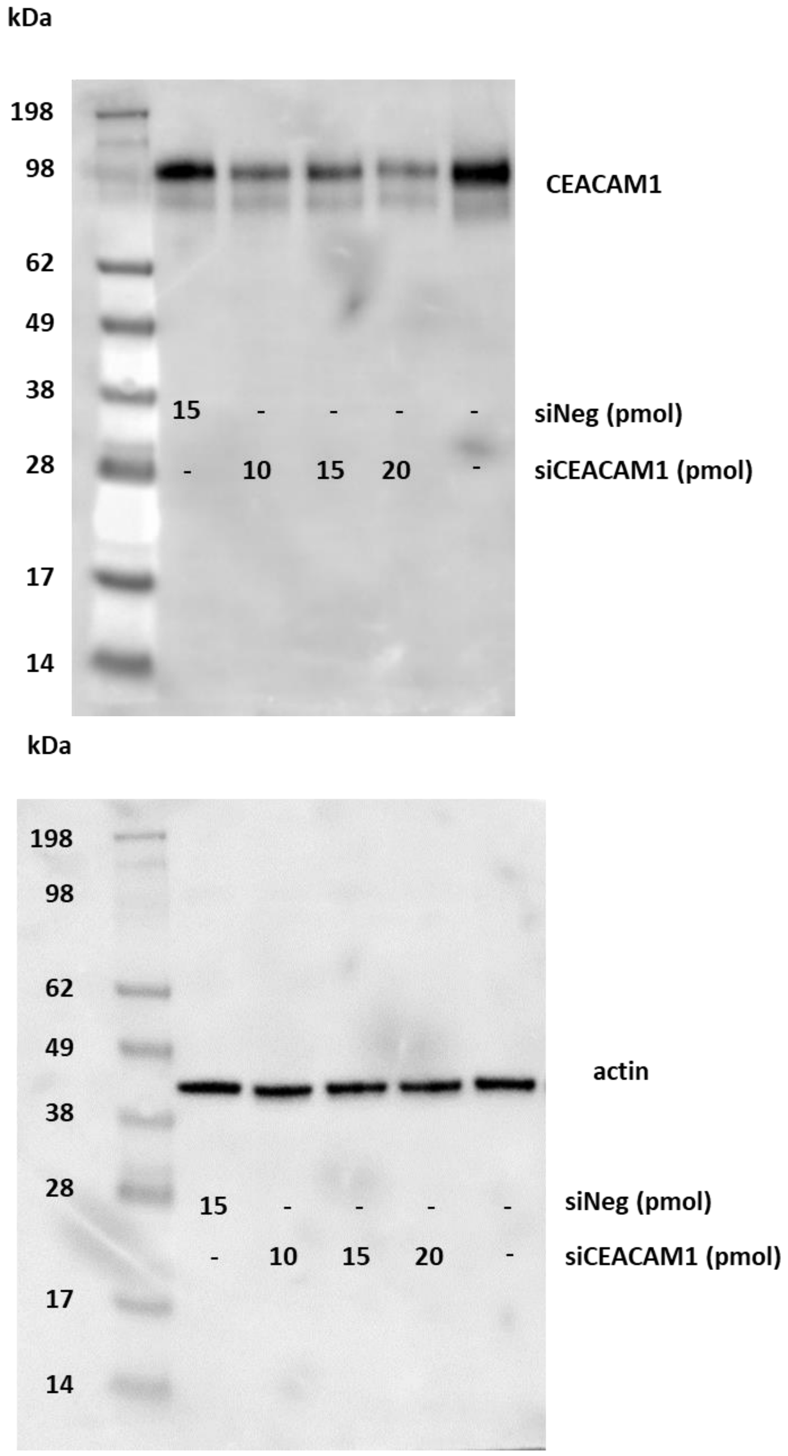

Supplement: Supplementary file 5 — Table S5 and Western blots [file 41598_2018_33605_MOESM5_ESM.pdf]
